# Annexin A2 as a target endothelial cell membrane autoantigen in Behçet's disease

**DOI:** 10.1038/srep08162

**Published:** 2015-02-02

**Authors:** Peng Chen, Hai Yan, Yaping Tian, Yiping Xun, Lili Shi, Ran Bao, Huai Zhang, Guangyu Chen, Chunhe Yang, Shutao Sun, Yajie Wang, Li Liu, Yabin Zhou, Chunyan Zhang, Xiaoxu Wang, Yongqiang Wen, Yongzhong Bian, Hongwu Du

**Affiliations:** 1 School of Chemistry and Biotechnology Engineering, University of Science and Technology Beijing, Beijing 100083, China; 2 Department of Clinical Biochemistry, Chinese PLA General Hospital, Beijing 100853, China; 3 ImmunoHunt Corporation, 139 Fengtai Rd, Beijing 100071, China; 4 Core Facility, Institute of Microbiology, Chinese Academy of Sciences, Beijing 100101, China; 5 Core Laboratory for Clinical Medical Research, Beijing Tiantan Hospital, Capital Medical University, Beijing 100050, China; 6 Department of Clinical Laboratory Diagnosis, Beijing Tiantan Hospital, Capital Medical University, Beijing 100050, China

## Abstract

Cell membrane proteins are believed to play a critical role in the pathogenesis of autoimmune diseases. However, few membrane autoantigens have been linked with Behçet's disease. Here, a cell-chip was performed to identify autoantibody target cells, and the suspected autoantigens were detected using immunoblotting. The amino acid sequences of the detected proteins were determined using LC-MALDI-TOF/TOF. Putative proteins were recombinantly expressed and purified, and a corresponding ELISA was developed and clinically validated using real clinical samples. It was found that a 36-kDa membrane protein - annexin A2 - was detected in approximately one-third of the patients' blood circulation. The immunohistochemistry results showed that annexin A2 was highly expressed in vascular endothelial cells. Moreover, vascular involvement was significantly higher in the anti-annexin A2 antibody-positive group versus the anti-annexin A2 antibody-negative group among all the clinical samples analyzed, indicating that annexin A2 is a novel endothelial cell membrane antigen involved in Behçet's disease.

Behçet's disease (BD) is a chronic multisystem vasculitis of unknown etiology^1^. This disease has global epidemiology but is more prevalent in regions spanning from East Asia (Japan, Korea and China) to the Mediterranean basin, including Turkey and the Middle Eastern countries^2^,^3^. Similar to other classical autoimmune diseases, such as systemic lupus erythematosus (SLE), Sjogren syndrome (SS) and rheumatoid arthritis (RA), BD also exhibits a diversity of clinical manifestations, indicating the co-existence of a large number of autoantigens^4^,^5^. In fact, efforts by other groups have led to the successful identification of some autoantigens, including the retinal S-antigen, IRBP, HSP70, a-tropomyosin, SBP, Mtch1, and annexin V^6^,^7^,^8^,^9^,^10^,^11^.

Moreover, vascular syndromes, which widely occur during BD progression, made researchers regard vascular endothelial cell target antigens as important factors in the pathogenesis of BD. Anti-endothelial cell antibodies (AECAs) have been detected in BD patients and have been proven to be associated with vasculitis symptoms^12^,^13^. Thus, scientists have emphasized the importance of endothelial cells and AECAs in the pathogenesis of BD. In the past decade, α-enolase and hnRNP-A2/B1 were successively identified in human dermal microvascular endothelial cells as self-antigens of BD^14^,^15^. Sip-1 and RLIP-76 were identified in human microvascular endothelial cells^16^,^17^, and kinectin was identified as a candidate autoantigen of BD in a bovine aortic endothelial cell line^18^. Prohibitin was also identified as a new endothelial cell autoantigen in our laboratory very recently using different systems biotechnology methods^19^. We believe that the combinatorial use of multiple high-throughput technologies might reveal new insight into the basic biology of autoimmune diseases, as multiplexed assay technologies at the molecular and cellular levels have enabled the identification of new biomarkers^20^,^21^.

These findings have provided clear information to understand the pathogenesis and have greatly expanded current knowledge of BD; however, many questions remain, particularly few of the specific self-antigens that primarily localized on the cell surfaces have been successfully identified. Clearly, membrane proteins represent a very important group of proteins and are involved in communication of cells to external stimuli^22^. Because intracellular proteins are far more likely to be the result of injury to tissue from some other primary process that the intracellular antigen does not target^23^. Autoantigens identified in autoimmune diseases that have membrane localization are more likely to play a key role in the original process of autoimmune diseases and further trigger autoimmunity. Therefore, the aim of this study was to identify cell membrane autoantigens of BD.

## Results

### EA.hy926 is a promising target to screen autoantigens

**Figure 1:**
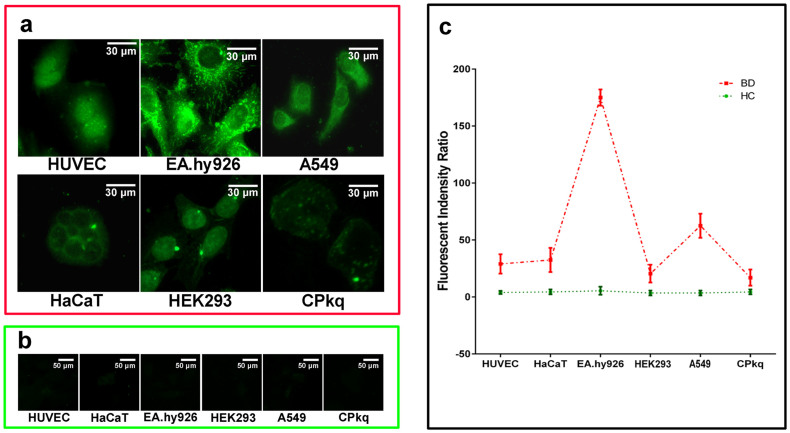
Indirect immunofluorescence assays on a cell-chip. Six cell lines were used to perform indirect immunofluorescence assays for selecting a candidate cell line on a cell-chip. (a) There were intense positive reactions in EA.hy926 cells with BD sera. (b) Healthy controls. (c) The cell fluorescence intensity without nucleus was obtained using Image J software (NIH, MD) and error bars are shown (mean with SD).

Six cell lines were cultured, developed as a cell-chip and used to prescreen the patients' sera to select a good candidate cell line for the identification of a membrane antigen. HUVEC showed positive binding signals to patient sera, which confirmed the presence of AECAs in BD patients. However, other cells also had abundant fluorescence signals, suggesting that these cells may be new autoantibody targets (Fig. 1a, b). Fluorescent differences and the fluorescence staining result without nucleus among the six cell lines were quantified using Image J software (Fig. 1c). The intense membrane staining of the EA.hy926 makes this cell line a good candidate for the identification of a membrane antigen.

### Identification of new autoantigens

**Figure 2:**
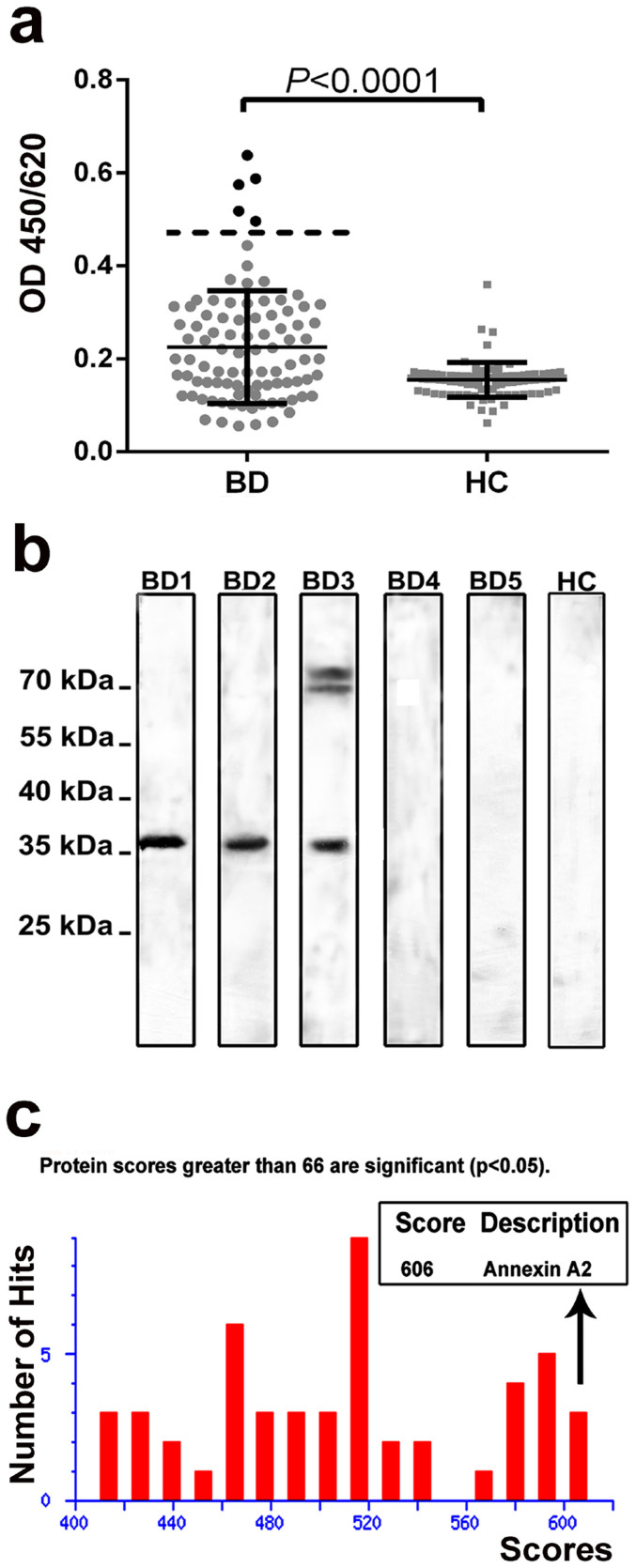
Specific autoantigens identified. (a) Membrane antigens-based ELISA was used to select BD patients who presented relatively higher optical density values of EA.hy926 membrane antigens for further testing. Error bars are shown (mean with SD). (b) Then protein immunoblot was performed to screen the putative binding target. Western blotting of EA.hy926 cell membrane extracts using the sera from BD patients showed a positive band around 36-kDa. Line 1-5 are sera from BD, line 6 is control serum from healthy donor. (c) The amino acid sequence of the positive band was identified by LC-MALDI-TOF-TOF mass spectrometry. BD, Behçet's disease. HC, healthy controls.

ELISAs of EA.hy926 membrane antigens with serum samples from 90 BD patients were investigated to select subjects containing large number of AECAs. Five subjects from all the BD patients that presented relatively higher optical density values were selected for further analysis (Fig. 2a). Then protein immunoblot was performed to display the putative binding target. Detection of autoantibodies binding to EA.hy926 antigens was observed and IgG autoantibodies to a 36-kDa cell antigen were detected in 3 of 5 BD patients, but no binding signal was detected for the healthy controls (Fig. 2b).

The newly identified 36-kDa autoantigen was cut from a polyacrylamide gel and digested with trypsin. The peptide fragments were identified using LC-MALDI-TOF-TOF mass spectrometry and then analyzed using the Mascot bioinformatics database to obtain the amino acid sequence of all the detective proteins. It was shown that the target protein shared the most significant sequence homology with annexin A2 (estimated molecular weight/pI, 38780/7.57; NCBI accession number, gi|18645167; Mascot score, 606; matched unique peptides, 19), which was accurately determined with over 95% confidence interval (>95 C.I.%) indicating credible results (Fig. 2c).

### Annexin A2 protein is a new autoantigen of BD

The selected target protein was obtained by recombinant DNA technology, and sequenced by mass spectrometry. Then using Western blotting, immunoprecipitation, and competitive immunofluorescence to confirm the protein is a real autoantigen of BD in succession.

**Figure 3:**
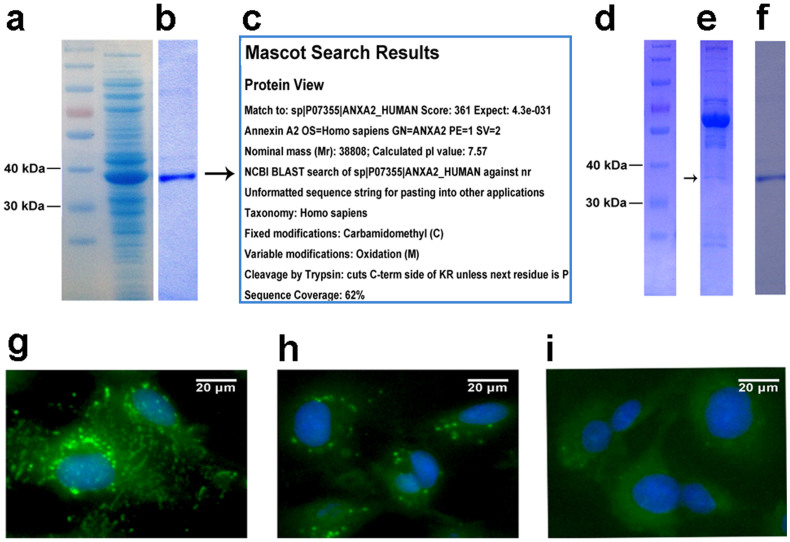
Expression, purification and verification of annexin A2 function by immunoprecipitation and competitive immunofluorescence assays. (a) Human annexin A2 protein was overexpressed in *E. coli* BL21 after IPTG-induced expression. (b) The annexin A2 protein was purified using Ni-NTA resin. (c) Verification of annexin A2 using mass spectrometry. (d) Protein marker. (e) Immunoprecipitation was performed to determine whether annexin A2 was a real autoantigen of BD. Annexin A2 was clearly present in the immunoprecipitates. (f) Purified annexin A2 protein served as a control in immunoprecipitation analysis. (g) The EA.hy926 staining pattern of sera from BD patients in competitive immunofluorescence assays which were used to analyze the inhibitory activity of the annexin A2 protein. (h) Pre-incubation of serum with annexin A2 resulted in inhibition of the staining. (i) EA.hy926 cells incubated with HC serum served here as a control.

First, expression and purification of the recombinant human annexin A2 protein were performed (Fig. 3a, b); the purified annexin A2 protein was confirmed using mass spectrometry (Fig. 3c) and Western blotting with purified mouse anti-human annexin A2 monoclonal antibodies in succession (data not shown).

Second, immunoprecipitation was performed to determine whether the detected antigen was a real autoantigen of BD. As shown in Fig. 3d–f, the band for the annexin A2 was clearly present in the immunoprecipitates, indicating that annexin A2 was indeed an autoantigen of BD that is present in patients' blood circulation.

Furthermore, competitive immunofluorescence assays were also used to analyze the inhibitory activity of the annexin A2 protein (Fig. 3 g–i). It was shown that the recombinant human annexin A2 protein could significantly reduce the binding activity of BD patient sera *in vitro*, which further proved that the anti-annexin A2 antibodies were found in the blood circulation of BD patients.

### The prevalence of anti-annexin A2 autoantibodies

**Figure 4:**
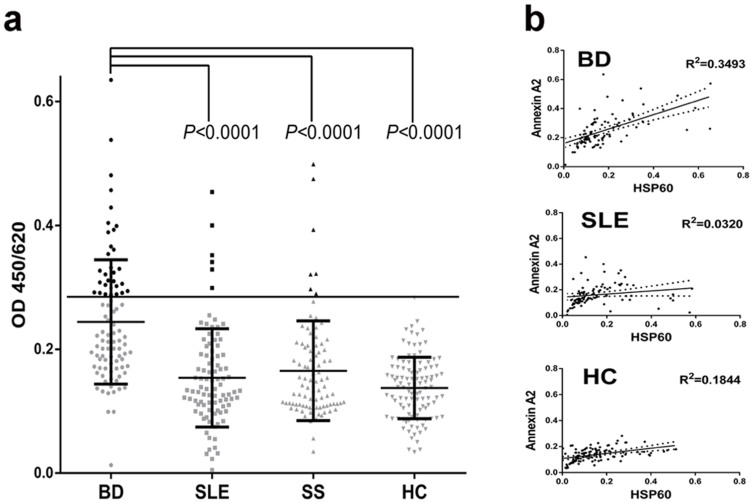
Reactivity of serum antibodies against annexin A2 and the relationship to HSP60. (a) Developed ELISA was used to detect the serum antibodies against annexin A2 in BD patients and controls. (b) The optical densities obtained from IgG autoantibodies against recombinant human annexin A2 were correlated with those against recombinant human HSP60. Trend lines were analyzed by linear regression with 95% confidence intervals (dotted lines) using SPSS (Version 17, Chicago, IL). BD, Behçet's disease. HC, healthy controls. SLE, systemic lupus erythematosus. SS, Sjogren syndrome.

To further investigate the role of annexin A2, the correspondence anti-annexin A2 ELISA was developed using the purified recombinant antigen. The anti-annexin A2 antibodies were detected in 31 of 90 BD patients (34%), 6 of 92 SLE patients (7%), 7 of 90 SS patients (8%), and 1 of 111 HC (<1%). The reactivity of sera IgG against human recombinant annexin A2 in BD patients was higher than that in SLE, SS and apparently healthy individuals (*P*<0.0001) (Fig. 4a).

### The correlation analysis of annexin A2 and HSP60 antibodies

The possible molecular interactions with annexin A2 during the process of BD were analyzed by ELISAs for the correlationship between annexin A2 and HSP60 (Fig. 4b). The optical densities obtained from sera against recombinant human annexin A2 were correlated with those against recombinant human HSP60 in BD patients (R^2^ = 0.3493, *P*<0.0001) and in SLE patients (R^2^ = 0.0320, *P* = 0.0814).

### Expression and localization of annexin A2 in human tissues

**Figure 5:**
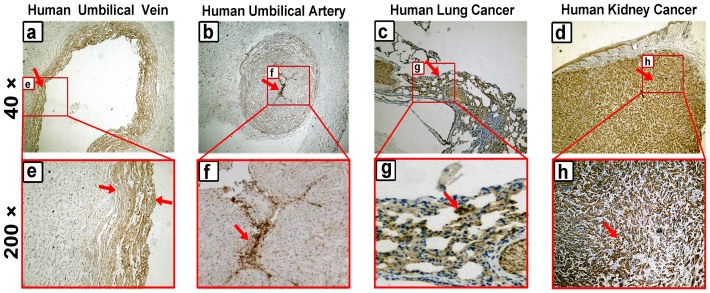
Expression and localization of annexin A2 in human tissues. Immunohistochemistry was used to analyze the differences in protein expression in human umbilical vein/artery, squamous cell lung cancer, and kidney cancer tissues. These tissues were incubated with anti-annexin A2 mAb and then (a–d) observed at ×40 for full-vision images and (e–h) at ×200 for partial vision images. The brown color represents positive identification with the anti-annexin A2 mAb.

**Figure 6:**
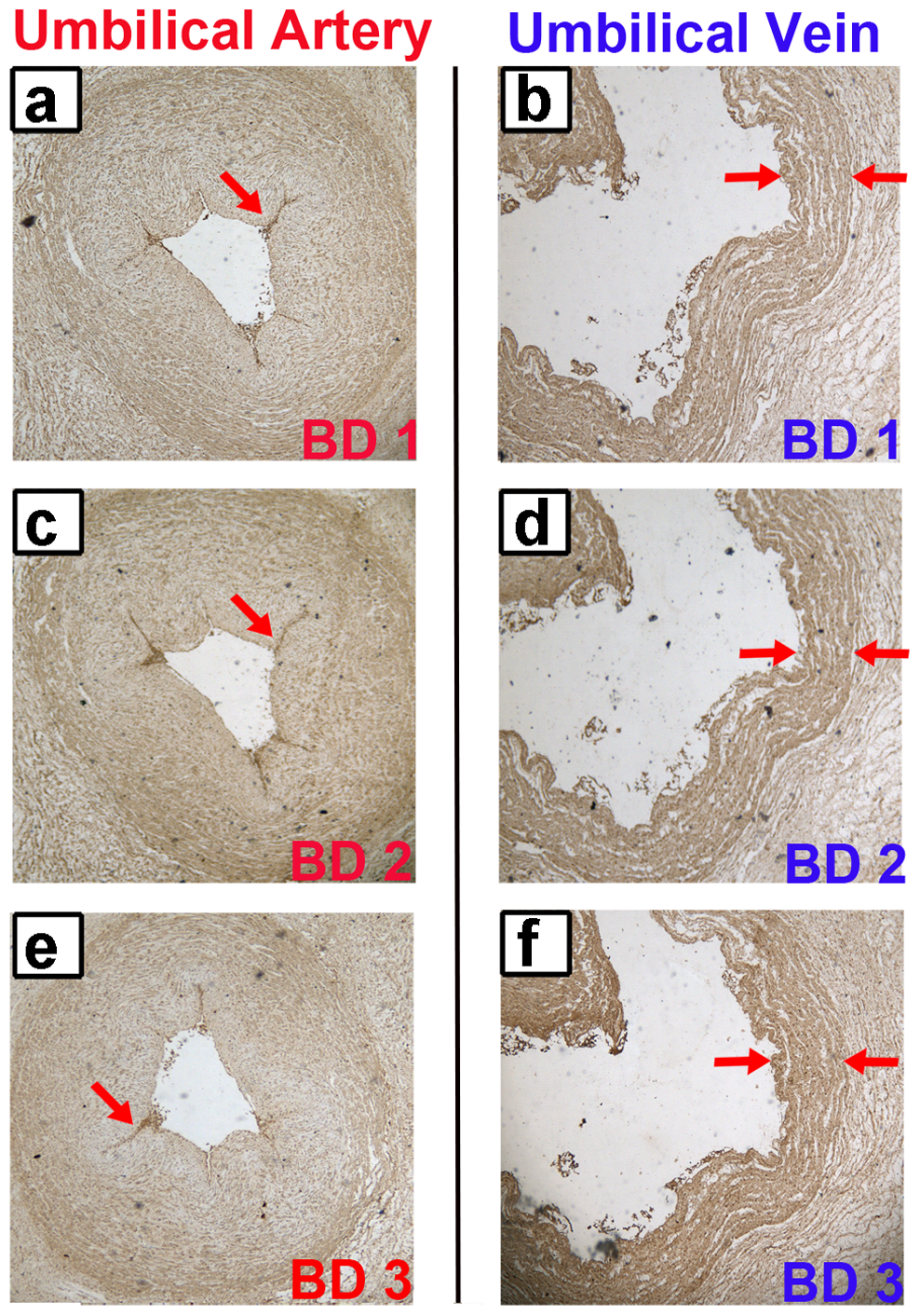
Staining of the BD autoantigens in human umbilical tissues. (a, b) The staining of the BD1 patient in human umbilical artery and vein was analyzed by immunohistochemistry method. (c, d) The staining of the BD2 patient in human umbilical artery and vein. (e, f) The staining of the BD3 patient in human umbilical artery and vein. The brown color represents positive identification with the BD patients' sera. This result confirmed the presence of AECAs in BD patients, and further indicated the annexin A2 as a real AECA autoantigen of BD.

Immunohistochemistry was used to analyze the differences in protein expression in human umbilical vein/artery, squamous cell lung cancer, and kidney cancer tissue samples. The results showed that annexin A2 was specifically expressed in human umbilical vein/artery vascular endothelial cells and not in other cells of the vessel (Fig. 5a, b). High expression of annexin A2 was clearly observed in human squamous cell lung cancer and kidney cancer tissue (Fig. 5c, d). These results are consistent with the data of Western blotting analysis, which also showed that the expression of annexin A2 in EA.hy926 cells was the highest among all the cells tested, followed by A549 cells and HUVEC (data not shown). The staining with commercial annexin A2 monoclonal antibodies in human umbilical vein/artery tissues (Fig. 6) was consistent with the anti-annexin A2 antibody-positive BD patients' sera. Put together, these results further show the annexin A2 as a real AECA autoantigen of BD.

### Clinical significance in BD patients

**Figure 7:**
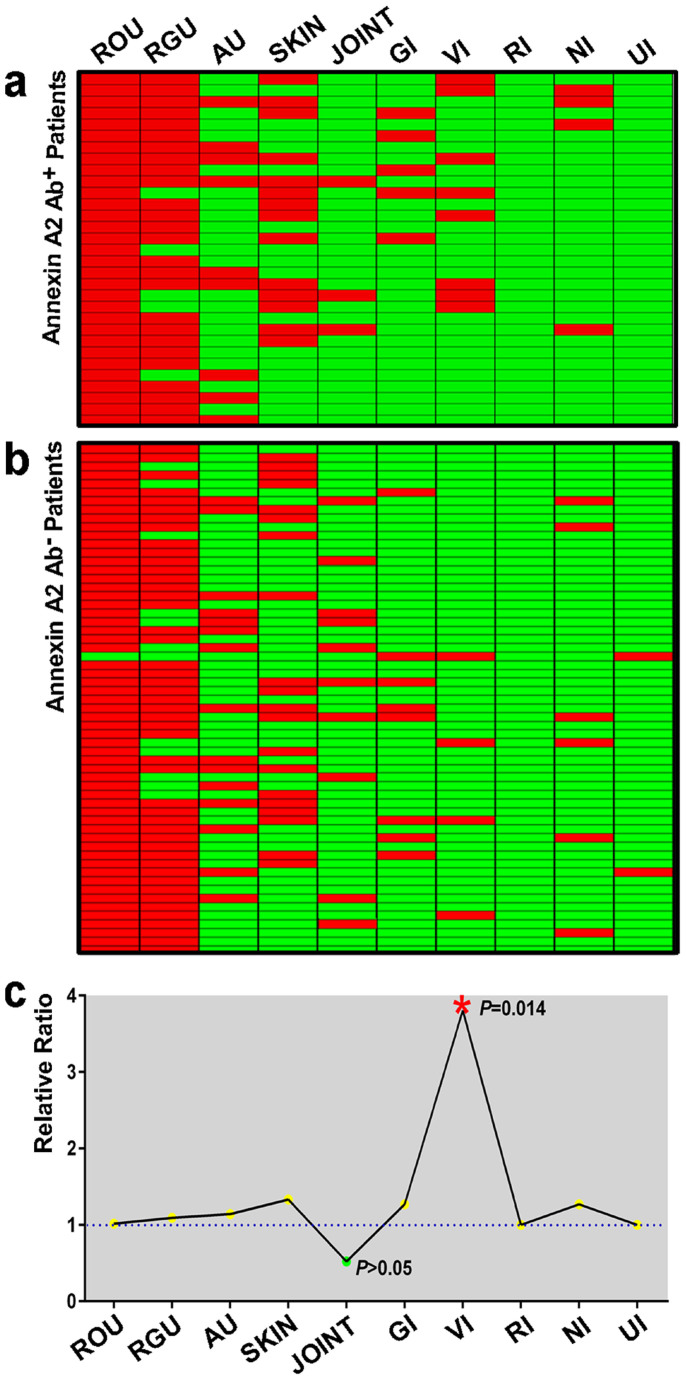
Relationship between clinical symptoms and anti-annexin A2 antibody. (a) Thirty-one BD patients of anti-annexin A2 antibody-positive were analyzed to determine the correlation to clinical information. Each square represents a patient. The red color represents the corresponding organ involved and green without. (b) The clinical information of fifty-nine anti-annexin A2 antibody-negative BD patient samples. (c) Comparing the clinical information between the anti-annexin A2 antibody-positive and -negative groups. The ratio was obtained from (Number^clinical+^/Number^positive^)/(Number^clinical+^/Number^negative^). The data were analyzed using SPSS software (Version 17, Chicago, IL). Recurrent oral ulcers (ROU), recurrent genital ulcers (RGU), skin lesions (SKIN), anterior uveitis (AU), vascular involvement (VI), gastrointestinal involvement (GI), nervous system involvement (NI), joint involvement (JOINT), respiratory system (RI) and urinary system (UI).

The correlationship between annexin A2 and clinical symptoms which involvement in BD patients was analyzed. Among the 90 BD patients, 31 subjects showed a positive reaction with the recombinant human annexin A2 protein. By comparing the clinical information between the 31 anti-annexin A2 antibody-positive and other 59 -negative subjects, vascular involvement was approximately four times higher in the anti-annexin A2 antibody-positive group. For all other clinical symptoms, no significant differences were observed. The detailed clinical information of all BD patients was analyzed and summarized in Fig. 7.

## Discussion

Primary vascular endothelial cells have been considered to be the original autoimmune target of BD, these cells are not necessarily the best candidate for the screening of blood circulation autoantibodies, which is partly due to their cell differentiation may cause variation in protein abundance in different situations^24^. In this study, a cell-chip was designed to identify autoantibody target cells and it was found that EA.hy926 cells possessed a significant binding ability, possibly because of their origin, which is a fused cell line with properties of both HUVEC and A549 cells^25^.

Annexin A2 is a phospholipid-binding protein that is widely distributed throughout cell membrane^26^. It is involved in several important biological processes, such as exocytosis and endocytosis, cell cycle regulation and immunoglobulin transport^27^. Annexin A2 overexpression has been described in many cancers, including liver, lung and kidney cancers^28^. This protein might play an important role in tumor cell proliferation and in the regulation of migration^29^, and it was identified as a vascular endothelial cell plasminogen receptor and plasminogen activator^30^ on the cell surface. Annexin A2 also plays a pivotal role in the process of thrombosis for antiphospholipid syndrome through mediating endothelial cell activation by anti-β2 glycoprotein antibodies^31^. Furthermore, deregulation of annexin A2 expression in acute promyelocytic leukemia is an important mechanism for bleeding diathesis^32^. Recent studies have indicated that annexin A2 may be a target for autoantibodies in SLE, SS, RA, and systemic vasculitis, and it has been hypothesized that anti-annexin A2 antibodies can induce thrombosis by activating endothelial cells and inhibiting plasmin generation^32^,^33^,^34^. Furthermore, another member of annexins has already been described, the serum anti-annexin V antibodies were significantly higher in neuro-BD patients compared to those without^11^. The homology analysis indicated that amino acid sequence of human annexin A2 protein had about 43% identity with annexin V. Our results related to the reactivity of anti-annexin A2 antibodies in SLE and SS are consistent with the reported literature, which further supports the reliability of this study.

In this study, annexin A2 was suggested to be a novel membrane autoantigen for BD and may be involved in the process of capillary injury during BD process. When comparing the clinical information between the anti-annexin A2 antibody-positive and -negative groups, vascular involvement was four times higher in the anti-annexin A2 antibody-positive group, indicating that annexin A2 may have a close relationship with vascular inflammation and damage.

A number of studies have shown that annexin A2 plays a role as a pathogenic receptor^35^,^36^,^37^, and most researchers believe that BD is related to infection by microorganisms. Sometimes, HSV (*herpes simplex virus*) considered being involved with the disease and the anti-HSV antibodies can be detected in the circulation serum of BD patients^38^. *Streptococcus sanguis* (*S. sanguis*) mainly inhabits the mucous membrane of the colon, mouth and throat, and the role of these organisms in BD immunopathogenesis has been reported^39^,^40^,^41^. The HSP65 of *S. sanguis* that is detected in the sera of BD patients is highly homologous with human HSP60^41^. Additionally, it was demonstrated that there is cross-reactivity between the antibodies from BD patient sera and *S. sanguis* or recombinant peptides of HSP65 from *S. sanguis*^42^,^43^. HSP60 can also elicit an immune response in humans, a response that although directed primarily against the microbial molecule^44^, providing a link between infection and pathogenesis of autoimmune diseases, as suggested for arthritis^45^,^46^,^47^ and multiple sclerosis^48^,^49^. Moreover, the expression of certain cytokines is up- or down-regulated after infection in BD patients. For example, some inflammatory factors, including IL-8 and TNF-α were up-regulated and IL-12 are down-regulated in patients with BD^50^, an increased level of IFN-γ was also observed in our patients with BD (data not shown).

In summary, annexin A2 was suggested as a specific vascular endothelial cell membrane autoantigen of BD. However, further investigations are necessary to elucidate the exact roles of annexin A2 in BD pathogenesis and long-term clinical monitoring effect of annexin A2 on more patients.

## Methods

### Subjects

In this study, serological criteria were evaluated through the assessment with a validation cohort of 383 subjects. The experimental group: 90 confirmed BD patients with an average age of 39 years (range: 16 to 64; 36 female and 54 male patients). The disease control group includes 92 SLE patients with an average age of 36 years (range: 18 to 59; 83 female and 9 male patients) and 90 SS patients with an average age of 51 years (range: 19 to 75; 87 female and 3 male patients). The healthy control group: 111 apparently healthy volunteers with an average age of 25 years (range: 21 to 33; 88 female and 23 male). The diagnosis of BD fulfilled the criteria proposed by the International Study Group for BD^51^. This study was approved by the ethical committee of Chinese PLA General Hospital, and each patient gave informed consent. Samples were collected from Sep. of 2012 to Feb. of 2014, dispensed and then stored at −80°C before further tests. The human tissues assays were performed at Beijing Tiantan Hospital. All methods in this study were carried out in accordance with the approved guidelines of Scientific Reports.

### Cell lines

The cell lines used in this study including human umbilical vein endothelial cells (HUVEC), a human umbilical vein cell line (EA.hy926), a human alveolar epithelial cell line (A549), a human embryonic kidney 293 cell line (HEK293), were purchased from American Type Culture Collection (Manassas, VA). The human immortalized non-tumorogenic keratinocyte cell line (HaCaT) was supplied by Cell Lines Service (Eppelheim, Germany). The EA.hy926, A549, HEK293, HaCaT were cultured in DMEM (HyClone, UT) containing 10% fetal bovine serum (HyClone, UT). HUVEC was cultured as described previously^19^. The oral epithelial cells were isolated from a healthy donor and labeled as CPkq according to his name. HUVEC served as control for the original generation of the vascular epithelial cells; HUVEC, EA.hy926, CPkq, HEK293 and HaCaT cells served as representatives of organ involvement; A549 cells served as a representative of a cancer cell line.

### Construction of the cell-chip

A multifunctional cell-chip was constructed based on our traditional microarray strategies^52^,^53^. A sample of each of the cultured cells was placed on cover slips, fixed with 4% paraformaldehyde, and then cut into fragments. The cover slips were then glued to microscope slides. Immunofluorescence assays were then carried out as follows. Sera from 90 BD patients were diluted 1:20 in PBS and incubated with the slides for 1 h at 37°C. After extensive washing, the slides were incubated with fluorescein isothiocyanate-conjugated goat-anti human IgG secondary antibody (ImmunoHunt, Beijing, China), which was diluted 1:150 in PBS, for 1 h at 37°C. The slides were then examined under fluorescence microscopy (AMG, Bothell, WA). The intensity was obtained using Image J software (NIH, MD) to display the intense fluorescence staining result without nucleus.

The competitive immunofluorescence assays was performed on the EA.hy926 cell line using serum from an anti-annexin A2 antibody-positive BD patient. For the inhibition assay, serum was pre-incubated with annexin A2 protein at 37°C for 1 h. After extensive washing, the slides were incubated with fluorescein isothiocyanate-conjugated goat-anti human IgG secondary antibody (ImmunoHunt, Beijing, China), which was diluted 1:150 in PBS, for 1 h at 37°C.

### Membrane antigens-based ELISA

The cell membrane proteins were extracted using a ProteoPrep® Membrane Extraction Kit (Sigma, MO) with 1% complete protease inhibitor cocktail (Sigma, MO). The extracts were dispensed and stored at -80°C until further use. The proteins (5 μg/mL) were used to coat the 96-well microplate (Corning, NY) overnight at 4°C. After three washes with 0.5% PBST, the plates were incubated with 10% goat serum for 1 h at 37°C. Sera from BD patients were diluted 1:100 with PBS and then added to each well. The plates were then incubated again for 1 h at 37°C. Horseradish-peroxidase-conjugated goat anti-human IgG (Invitrogen, CA) was diluted 1:10000 in PBS with divalent cations and then incubated for 30 min at 37°C. The amount of bound antibody was quantified colorimetrically by adding tetramethylbenzidine as a substrate, and the plates were read spectrophotometrically at 450/620 nm on an ELISA reader (Tecan, Hombrechtikon, Switzerland).

### Western blotting

The Western blotting was performed as described elsewhere^54^ with slight modifications. The cell membrane proteins extracted using a ProteoPrep® Membrane Extraction Kit (Sigma, MO) with 1% complete protease inhibitor cocktail (Sigma, MO). The extracts were dispensed and stored at −80°C until further use. Cell lysates were loaded into the wells of a 12% polyacrylamide gel and separated. The gel was then transferred onto polyvinylidene fluoride membranes (PVDF; Merck Millipore, MA) that had been washed twice with ultrapure water. The membranes were then blocked with 5% nonfat milk in PBS at 4°C overnight and then incubated with 5 BD sera that had been selected due to their relatively higher optical density values (1:500 dilution) or sera from random healthy controls at 4°C for 12 h. The membranes were extensively washed four times with 5% PBST buffer to remove unbound antibodies. Finally, they were incubated with horseradish-peroxidase-conjugated goat anti-human IgG (ImmunoHunt, Beijing, China) for 1 h at 37°C, and ECL detection was carried out in accordance with the product instructions (Applygen, Beijing, China).

### In-gel digestion and mass spectrometry

In-gel digestion and mass spectrometry were performed as described in detail elsewhere^55^. Briefly, the excised gel pieces were destained with 25 mM NH_4_HCO_3_ and 50% acetonitrile and dried by vacuum centrifugation. Then, 10 mM dithiotreitol in 25 mM NH_4_HCO_3_ was added to cover the gel pieces, and the samples were reduced for 2 h at 37°C. After cooling to room temperature, the DTT solution was replaced by roughly the same volume of 55 mM iodoacetamide in 25 mM NH_4_HCO_3_ and incubated for 45 min at room temperature in the dark. The gel pieces were washed with 25 mM NH_4_HCO_3_ in 50% acetonitrile for 10 min. After the liquid phase was removed, the gel pieces were completely dried by a vacuum concentrator. The dried gel pieces were then covered with 20 μL of 0.05 M NH_4_HCO_3_ buffer containing trypsin (Sigma, MO), and digestion was performed overnight at 37°C. The excised 36-kDa protein band was identified using a 5800 Proteomics Analyzer LC-MALDI-TOF/TOF mass spectrometer (Applied Biosystems, Foster City, CA). Mass spectrometry data were analyzed with Mascot bioinformatics database search engine (Matrix Sciences, London, UK).

### Protein expression and purification

The procedure of protein expression and purification was performed as our routine method^56^. In brief, total RNA was isolated from EA.hy926 cells using TRIzol reagent (Invitrogen, CA). RT-PCR was carried out according to the manufacturer's instructions (Fermentas, MD). Target proteins were overexpressed in *E. coli* BL21, followed by purification of the recombinant proteins using Ni-NTA resin (Qiagen, Hilden, Germany). The concentration of protein was determined using a BCA assay kit (Biosynthesis Biotechnology, Beijing, China). Purified recombinant protein was confirmed by mass spectrometry and Western blotting with mouse anti-human monoclonal antibody (Proteintech, Chicago, IL).

### Immunoprecipitation

Immunoprecipitation was performed as previously described^57^. The putative autoantigen (5 μg) was incubated with 5 μL mixed sera (equal volumes from three positive BD patients in Western blotting detection) at 4°C overnight. Thereafter, protein A-Sepharose beads (Sigma, MO) that had been washed with PBS were added and mixed overnight at 4°C. The immunoprecipitates were washed three times in 200 μL PBS. Then, the immunoprecipitates were suspended in sample loading buffer and resolved by 12% SDS-PAGE.

### Development of an ELISA

ELISAs with human recombinant proteins were performed as described previously^54^. In brief, 1 μg/mL recombinant human annexin A2 or HSP60 (Sino biological, Beijing, China) were used to coat the 96-well microplate (Coring, NY) overnight at 4°C. After three washes with 0.5% PBST, each well was blocked with 200 µL 5% goat serum for 2 h at 37°C. Then, the plate was incubated with 100 µL sera from patients or healthy controls, which were diluted 1:100 in PBS, for 2 h at 37°C. Three washes later, 100 µL of goat anti-human IgG/HRP (ImmunoHunt, Beijing, China) was added to each well, and the plate was incubated for an additional 1 h at 37°C. Following three washes, 50 µL tetramethylbenzidine (TMB) A (0.1 M citric acid, 0.2 M NA_2_HPO_4_, 0.6 g hydroperite/L) and 50 µL TMB B (5 mM citric acid, 0.4 mM EDTA-NA_2_, 0.2 g TMB/L) were added to each well. Then, the plate was incubated in the dark at room temperature for 4 min, and the reaction was stopped by adding 50 µL of 2 M H_2_SO_4_. The absorbance of each well was measured with a plate reader at 450 nm (Tecan, Hombrechtikon, Switzerland).

### Immunohistochemistry

The immunohistochemistry protocols, with slight modification, have been previously described^58^. The human umbilical vein/artery, squamous cell lung cancer and kidney cancer tissue samples were treated with 4% paraformaldehyde overnight at 4°C, and the fixed tissues were embedded in paraffin wax. Five-micrometer-thick paraffin sections were placed on glass slides, baked for 60 min at 60°C, incubated for 10 min in xylene, and finally washed with PBS. To inhibit endogenous peroxidase, the sections were first incubated in 3% H_2_O_2_ in methanol for 20 min at room temperature. Then, the sections were boiled in sodium citrate-hydrochloric acid solution for 10 min and incubated in goat serum for 30 min at 37°C. The sections were washed with PBS and then incubated overnight at 37°C with mouse anti-human annexin A2 monoclonal antibody (Proteintech, Chicago, IL), which had been diluted in PBS (1:100). The sections were washed again with PBS to remove unbound antibodies and then incubated with HRP-labeled goat anti-mouse IgG (ImmunoHunt, Beijing, China). The sections were washed again and then incubated with 3, 3-diaminobenzidine (DAB) to develop peroxidase activity. Counter-staining was performed with hematoxylin. The sections were dehydrated in gradient ethanol (approximately 2 min at each concentration), placed in xylene for 5 min, and then observed with a microscope (Olympus, Tokyo, Japan).

### Statistical analysis

The t-test statistics, Spearman's correlation coefficients, Fisher's exact text, and chi-squared test were analyzed using SPSS software (Version 17, IL). *P* values less than 0.05 were considered significant. The critical point for positive definition was a number with a higher value than that of the healthy controls (Mean + 3 SD).

## Author Contributions

H.W.D., Y.P.T., Y.Q.W., Y.Z.B., P.C. designed the research; P.C., H.Y., Y.P.X., L.L.S., R.B., H.Z., C.H.Y., S.S.S., Y.J.W., L.L., Y.B.Z., performed the experiments and analyzed the data; P.C., C.Y.Z., X.X.W., H.W.D., G.Y.C. wrote the manuscript; all authors discussed the results and commented on the manuscript.
